# The British Hospitals Association Conference

**Published:** 1922-07

**Authors:** 


### THE BRITISH HOSPITALS ASSOCIATION CONFERENCE.

IN our June issue we published the paper on "The Change in Hospital Finance," read by Sir Alan Garrett Anderson on the first day of the British Hospitals Association Conference at Liverpool. As the Conference was sitting when we went to press, we were not able to report its proceedings.

In an opening speech, the President, Sir Arthur Stanley, recalled an interview which he and others had about three years ago with Dr. Addison, the then Minister of Health, when he found himself in a minority of one. Every other speaker advocated the complete or partial placing of the hospitals on the rates. He had the courage to assert that if hospital authorities could tide over the next three or four years, the voluntary system would re-assert itself and they could trust to the generous public to give them the money they required. He was beginning now to think that he was right. The public, he believed, had determined that their voluntary hospitals were not to go. The fact that they had had to stir themselves and make an effort was probably the best thing that could have happened.

The discussion on Sir Alan Garrett Anderson's paper (HOSPITAL AND HEALTH REVIEW, June, p. 249) was opened by Mr. H. Wade Deacon, Chairman of the Liverpool Royal Infirmary, and Chairman of the Council of the B.H.A., who said that Sir Alan was no doubt right in his view that money could be raised by an organisation which would deduct contributions from working men at a rate of a few pence a week. It was a very open question as to what would be a proper deduction. Those responsible for the hospitals should watch the liability they were incurring. If a man who contributed so much a week believed that he thereby had a right to treatment for himself and his wife and children, the hospitals would be undertaking a great responsibility. In many parts of the country the Hospital Saturday Fund subscribed largely to the hospitals, but in most towns, as far as he knew, contribution to such a fund did not confer any distinctive right of admission to hospital. In Liverpool the hospitals made no definite charge, but patients were asked to contribute according to their means. Speaking broadly, it was found that they were only too glad to contribute once they understood the need.

Several delegates then gave particulars of the contribution schemes in force in their centres. Dr. J. Maxtone Thom, of the Glasgow Royal Infirmary, said that in Glasgow and district a scheme had worked well for some years under which the workers contributed 1d. per week on wages below 30s., 1½d. on wages between 30s. and 40s., 2d. between 40s. and 60s., 3d. between 60s. and 90s., 4d. between 90s. and 120s., 5d. between 120s. and 140s., 6d. on 140s. and over.

Mr. Hampson, of Sheffield, reported a similar scheme, the workers paying 1d. in the pound on their wages. The employers added one-third of the total contributed by the workers. In the five months ended in May of this year something like £21,000 went to the hospitals under this scheme, despite bad trade.

Mr. J. A. Cowley (Northwich) said they must be be careful lest, in introducing schemes of this kind, they discouraged people from supporting voluntary funds which hitherto had been the backbone of the hospitals. There was a reservoir which ought to be tapped by voluntary hospitals, in the shape of the surplus funds of approved societies.

Mr. Arthur Griffiths gave details of the agricultural scheme introduced by the East Suffolk and Ipswich Hospital, where the wage-earners in agricultural pursuits gave 1d. per week each and the farmer gave a sum equal to the total of the employee's contributions. He drew attention to the decline since 1914 of legacies to provincial hospitals, and thought that in the absence of a Central Provincial Hospital Fund, many large legacies from the country found their way into the coffers of the King's Fund for London. He therefore advocated the inauguration of a provincial fund, on the lines of the King's Fund, so that legacies bequeathed to such a fund could be allocated to the hospitals in the county where testator had been domiciled.

Mr. W. H. Harper (Wolverhampton) said that the working classes should be taught to give during the time of good health, and should not be worried about financial matters when they were lying in hospital.

Mr. J. H. Shaw (Southport) pointed to the scheme of hospital contributions which had been evolved in Southport among the gas-workers and railwaymen.

Mr. Mark Rathbone (Liverpool Maternity Hospital) was anxious to know how the trade unions regarded the London scheme. He looked upon a scheme of universal contributions as the easiest way out of the financial difficulties, although he dissented from the view that contributions to the hospital should imply a right to special treatment.

The President commented on the "wonderful assistance" which the wage-earners were giving. "It makes one realise," he said, "what we suspected before—that we in London are very much behindhand in all these matters." He added that the voluntary system had already saved itself in the country generally, if not yet in the big towns.

In the afternoon Mr. W. Thelwall Thomas, of the Liverpool Royal Infirmary, read the paper on "The Voluntary Hospital: a Retrospect and a Prospect," which we publish on page 290 of this issue. The question whether hospitals should make claims upon approved societies in respect of their services to insured persons was discussed at some length.

Mr. H. Wade Deacon said that although £250,000 had been set aside by approved societies for distribution to hospitals, the Hospitals Commission was informed that the hospitals were not claiming anything like the amount anticipated. Unless applications increased, the £250,000 would not be used.

The Rev. G. B. Cronshaw, of the Radcliffe Infirmary, Oxford, said his hospital would not claim this money because, if they did, that would give the approved societies a right to demand some control of the hospitals. That was a thing he dreaded.

Mr. Griffiths (Ipswich) said we were gradually converging on the American system of hospital maintenance, because the people of this country no longer wanted the stigma of "the indigent poor" to be attached to them when they needed hospital treatment.

Mr. G. Q. Roberts (St. Thomas's Hospital) said that the National Health Insurance Act did not provide for the treatment of cases of serious illness when they came into hospital. The hospitals did not confine their work to the sick poor; they had for 30 years been treating those classes of the community who could not afford to pay the heavy costs of serious operations, or of the treatment of a disease which was not easily discernible to the general practitioner. Thirty years ago it was said that the poor were getting treatment in hospital which no better-circumstanced people with less than £5,000 a year could get in their private houses, and it was probably even truer to-day. He agreed heartily as to the willingness of the working man to contribute to hospitals. He had served on a committee of that association which had met representatives of the leading approved societies, to formulate a scheme whereby the hospitals would get full benefit of surplus funds. The managers of those societies were anxious to do all they could for the hospitals, but there were no vast sums for distribution He was very much surprised to hear that the sum available was not being distributed because the hospitals were not applying for it. He could not see why that money should not be accepted, if not for the maintenance of patients, at least for building improvements and the general development of the hospitals.

Mr. H. E. Wild (Wallasey) thought that the salvation of voluntary hospitals would be found in the erection of private wards, in which patients would be treated at a fixed charge.

Mr. J. H. Shaw (Southport) said many of the panel doctors of the country had grown financially fat at the expense of hospitals.

Complaints were made of the difficulty of finding out from illiterate patients what approved societies they belonged to, which hindered the presentation of claims.

At the close of the debate Sir Arthur Stanley asked how many of those present wished the Association to continue negotiating with the approved societies with a view to getting payment in some form? Every hand was held up. How many had actually applied for payment? About half those present indicated that they had done so. Sir Arthur then explained that the Council of the Association would take up with the approved societies any difficulties that the hospitals might have.

On the second day of the Conference, in moving the adoption of the Association's annual report, Sir Arthur Stanley expressed the opinion that if the recommendations of Lord Cave's Commission had been carried out in their entirety the hospitals would have been out of the wood by this time. As to nurses' wages and salaries, a scale had been realised, but it was not possible for all the hospitals to adopt it as a minimum scale at present.

Mr. Wade Deacon said the Hospitals Commission had to deal with half a million of money, which it was estimated was one-half of the total deficit of the hospitals for 1921. They had up to now distributed £103,000 in London and £39,000 in the rest of the country. The London grants had been chiefly emergency grants to save the closing of beds or to cause beds to be reopened. The applications from the country were not as numerous as might have been expected.

After the re-election of Sir Arthur Stanley as president of the Association, Mr. Frank Hazell, general superintendent and secretary of the Manchester Royal Infirmary, read a paper on "Hospital Economies," which we hope to publish next month, advocating, as a principal measure, the establishment of a central provincial hospital fund for the benefit of the 650 provincial hospitals.

In the discussion which followed, the installation of oil fuel in hospitals was recommended by Mr. G. Q. Roberts, who said that at St. Thomas's Hospital they had effected in eight months a saving of £1,850 by substituting oil fuel for coal, and the efficiency secured had been most remarkable.

Col. J. J. Shute (Liverpool) thought that the system of co-operative buying had not been tried to the fullest extent. There was a great deal in the principle that might be of benefit to hospitals, large and small. He believed it would be possible in large towns to have a central stores, the personnel of which would be directly controlled by the hospitals, and the buyer must be thoroughly conversant with the movement of the markets.

The Chairman asked whether delegates were prepared to give any expression of opinion as to the establishment of a provincial central fund, and several spoke in its favour. Viscount Hambleden thought the initiation of such a fund would have the effect of increasing co-operation between the provincial hospitals. A motion was adopted, with three dissentients, approving of the formation of a fund on the lines of King Edward's Fund for London, and asking the Council of the Association to take the matter into active consideration.

Sir Arthur Stanley said definite instructions had now been given that the members wished the Council to pursue their negotiations in regard to approved societies contributing to hospitals, and further to consider the possibility of setting up a central fund for provincial hospitals. If they had this central fund he thought one of the principal rules should be the distribution of money according to the work done, and not necessarily by the size of a hospital overdraft.

Charming hospitality was shown to those attending the Conference by the Local Committee supervising the arrangements, under the chairmanship of Mr. H. Wade Deacon. On May 26 the delegates were the guests of the Lord Mayor of Liverpool at tea at the Town Hall, and in the evening attended a dinner at the Exchange Station Hotel.

